# Investigating the state of physiologically based kinetic modelling practices and challenges associated with gaining regulatory acceptance of model applications

**DOI:** 10.1016/j.yrtph.2017.08.019

**Published:** 2017-11

**Authors:** Alicia Paini, Jeremy A. Leonard, Tomas Kliment, Yu-Mei Tan, Andrew Worth

**Affiliations:** a European Commission, Joint Research Centre, Directorate Health, Consumers and Reference Materials, Via E Fermi 2749, 21027 Ispra, Italy; b Oak Ridge Institute for Science and Education, Oak Ridge, TN 37831, USA; c Klimeto, Juzna 4, Roznava, Slovakia; d U.S. Environmental Protection Agency, National Research Laboratory, Research Triangle Park, NC 27709, USA

**Keywords:** PBK models, Physiologically based kinetic models, Regulatory, Toxicology, Risk assessment, Alternatives methods

## Abstract

Physiologically based kinetic (PBK) models are used widely throughout a number of working sectors, including academia and industry, to provide insight into the dosimetry related to observed adverse health effects in humans and other species. Use of these models has increased over the last several decades, especially in conjunction with emerging alternative methods to animal testing, such as *in vitro* studies and data-driven *in silico* quantitative-structure-activity-relationship (QSAR) predictions. Experimental information derived from these new approach methods can be used as input for model parameters and allows for increased confidence in models for chemicals that did not have *in vivo* data for model calibration. Despite significant advancements in good modelling practice (GMP) for model development and evaluation, there remains some reluctance among regulatory agencies to use such models during the risk assessment process. Here, the results of a survey disseminated to the modelling community are presented in order to inform the frequency of use and applications of PBK models in science and regulatory submission. Additionally, the survey was designed to identify a network of investigators involved in PBK modelling and knowledgeable of GMP so that they might be contacted in the future for peer review of PBK models, especially in regards to vetting the models to such a degree as to gain a greater acceptance for regulatory purposes.

## Highlights

• Physiologically Based kinetic (PBK) models are used widely in academia, industry, and government.
• Good modelling practice (GMP) for model development and evaluation continues to expand.
• Further guidance for establishing GMP is called for.
• There remains some reluctance among regulatory agencies to use PBK models.
• The next generation of PBK models could be developed using only data from *in vitro* and *in silico* methods.

## Graphical abstract

**Figure:**
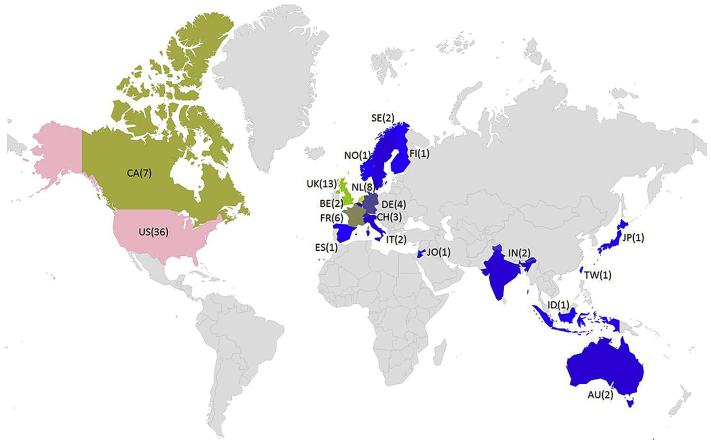

## 1 Introduction

**Fig. 1:**
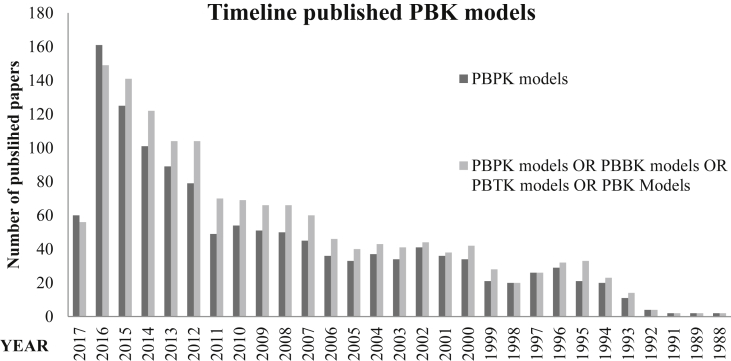
Number of papers published per year in the last 30 years. The search was conducted using PubMed on the 08th of April 2017, with key words including “PBPK model” only, or a set of keywords including the string “PBPK models OR PBBK models OR PBTK models OR PBK Models”. The year 2017 represents only papers published in the first 3 months.

Physiologically based kinetic (PBK^1^) models describe the body as a set of interconnected compartments that represent plasma and various organs, and characterize a chemical's fate within the body in regards to pharmacokinetic properties including absorption, distribution, metabolism and elimination (ADME). The development and use of PBK models have significantly increased over the last two decades, as is reflected in the rise of published literature referencing PBK models (Fig. 1). A wide variety of institutions have expressed a high degree of interest in PBK model applications, including academia, regulatory agencies, pharmaceutical and chemical industries (Schuck et al., 2015). PBK models have been deemed an effective means to aid in *in vitro* to *in vivo* extrapolation (IVIVE) (Blaauboer, 2010, Kramer et al., 2015), route to route extrapolation (Bessems et al., 2017), high to low dose, and inter- and intra-species extrapolations (Punt et al., 2016).

The European Union Reference Laboratory for Alternatives to Animal Testing (EURL ECVAM), which is part of the European Commission, published its Toxicokinetic Strategy (2015)^2^ outlining a plan for identifying opportunities to apply new approach methods (NAM) to generate and more effectively use toxicokinetic data. The central feature of this strategy is using PBK models to integrate ADME data and to predict whole-body toxicokinetic behaviours. To facilitate the acceptance of PBK models for which development is based solely on non-animal data, good modelling practice (GMP) focusing on this new generation of PBK models is required for further development and recognition at an international level. As a starting point, GMP should include relevant information from existing guidance documents (WHO/IPCS (World Health Organization. International Programme on Chemical Safety), 2010, US EPA, 2006, EMA European Medicine Agency, 2016, US FDA, 2016). For example, development of PBK models can be accomplished through a six-step approach (Rietjens et al., 2011), regardless of the use of conventional methods or NAM to parameterize models. Briefly, these steps include (i) defining a simplified representation of the biological system for which model compartments can be included (i.e., a conceptual model); (ii) translating the conceptual model into a mathematical model by formulating a differential equation for each compartment; (iii) defining equation parameters as values derived either from literature or from experiments; (iv) solving the equations by calculating the concentrations of relevant compounds and their metabolites in the specific compartments, or the extent of their adverse reactions with the toxicological receptor; (v) evaluating model performance, with adjustments and improvements to the model when necessary; and (vi) using simulations to make predictions (Rietjens et al., 2011). The process involving each of these six steps should be transparent and documented. In addition, GMP may involve the development of standard reporting formats, which is equivalent in purpose to the QSAR Model Reporting Format (QMRF) and QSAR Prediction Reporting Format (QPRF), for presenting sufficient details of model construction and application. Such detailed reporting will aid kinetic modellers in accounting for all the necessary components that constitute a proper model and is expected to facilitate acceptance of kinetic modelling approaches by decision makers. Finally, the formation of a panel comprised of international PBK modelling experts and a knowledgeable group of peer-reviewers would not only expedite the drafting of GMP, but also promote future applications of PBK models in regulatory risk assessment.

As toxicity testing and risk assessment shift more towards approaches involving NAMs such as *in vitro* techniques, PBK models are being used more frequently to convert *in vitro* points of departure (PoDs) to *in vivo* exposure (i.e., reverse dosimetry) for risk screening. Several examples are outlined in the review by Punt et al. (2011), including those for neurotoxicity (DeJongh et al., 1999, Forsby and Blaauboer, 2007), acute and repeated dose toxicity (Gubbels-van Hal et al., 2005, Rotroff et al., 2010, Pery et al., 2013, Gajewska et al., 2015), developmental toxicity (Verwei et al., 2006, Louisse et al., 2010), and genotoxicity (Paini et al., 2010). Among these examples, Paini et al. (2010) converted a threshold for a molecular initiating event (e.g., DNA binding) to an external exposure; Pery et al. (2013) and Gajewska et al. (2015) linked *in vitro* cell viability to an external dose; Wetmore et al., 2012a, Wetmore et al., 2012b incorporated *in vitro* metabolism measurements in IVIVE and reverse dosimetry to estimate external doses that are relevant to *in vitro* PoDs.

One of the major limitations for a broader application of PBK models highlighted by Punt et al. (2011) is that development of new PBK models can be labour intensive processes that require generation of a large range of data through *in vitro*, *in silico*, or *in vivo* analyses to construct and parameterize a model. A more efficient approach is starting with the structure of a previously developed, well-parameterized, and thoroughly-vetted model for a close analogue; and adjusting chemical-specific parameters based on *in vitro* or *in silico* data (e.g., metabolism rates, absorption rates, partition coefficients between blood and tissues) (Lu et al., 2016). Such approach also directs the modellers to obtain necessary *in vitro* or *in silico* data that are relevant to a specific chemical.

In order to facilitate acceptance and use of the new generation of PBK models, which rely on non-animal data, in the regulatory domain, experts were invited by EURL ECVAM to participate in a workshop on “Physiologically-based kinetic modelling in risk assessment – reaching a whole new level in regulatory decision-making” (Ispra, Italy, November 16–17, 2016) to identify challenges in (i) applying PBK modelling to support regulatory decision making; (ii) constructing PBK models without the use of *in vivo* kinetic and dynamic data, instead, relying solely on *in vitro* or *in silico* methods; and (iii) assessing model credibility and validity. The workshop participants concluded that an updated GMP requires inclusion of strengths and limitations encountered when parameterizing PBK models using *in vitro* measurements and *in silico* predictions, as well as processes for evaluating these types of PBK models to support regulatory decision-making. Other outcomes of the workshop and recommendations of the workshop participants are summarized by Paini et al. (2017). Prior to the workshop, in October 2016, the organizers sent invited experts a questionnaire containing 11 questions, to understand the use of PBK models from these experts (Paini et al., 2017). Due to the agreement among the experts that this exercise should be expanded to a broader level, the survey entitled “Applications of Physiologically Based Kinetic (PBK) models in science and regulatory submission” was created and extended to a larger, international PBK modelling and user community. The objective of this article is to outline and report the results from this survey to provide insight into the current applications of PBK models in research and regulatory communities, as well as challenges in regards to applying and evaluating PBK models that are constructed using data generated by NAMs. The survey findings and our analyses of the results provide valuable information on current practices in model development, application, and evaluation using a variety of modelling platforms. This information will be used to identify the key elements that should be included in the updated guidance on GMP for PBK models designed to support regulatory risk assessment applications.

## 2 Methodology

A survey was created by the European Commission - Joint Research Centre (JRC), EURL-ECVAM (https://eurl-ecvam.jrc.ec.europa.eu/) to understand the frequency of use and applications of PBK models in research and regulatory communities. The survey was published on the 11th of January 2017 and remained opened for 7 weeks until the 28th of February. The survey was made available to the participants at the workshop entitled “Applying exposure science to increase the utility of non-animal data in efficacy and safety testing” hosted by the National Centre for the Replacement Refinement & Reduction of Animals in Research (NC3R; https://www.nc3rs.org.uk/) in February 2017. To increase the size of the participant pool, the survey was then reopened for the members of the Biological Modelling Speciality Session of the Society of Toxicology (SOT-BMSS; https://www.toxicology.org/groups/ss/BMSS/index.asp) from the 14th to the 30th of April, 2017.

The survey was conducted using an online questionnaire via the European tool EUSurvey (https://ec.europa.eu/eusurvey/home/welcome) and was comprised of 18 primary questions; 14 with YES/NO or multiple choice answers and 4 in free text format. Some of the YES/NO questions were expandable to a secondary related question, depending on a participant's reply. In some cases, additional comments could be entered as free text. The survey was grouped into four main areas: PBK model construction, evaluation, applications, and acceptance by regulatory agencies. In addition, the country of residence, professional affiliation, and expertise of the respondents were collected by the survey. The full questionnaire is available in the supplementary material. For the purposes of this analysis, responses to the questionnaire were aggregated and individual responses were kept anonymous.

The target group for this survey was those involved in PBK model development and/or application in both research and regulatory communities, starting with approximately 200 individuals who had publications in PBK modelling. No individuals could be identified for Brazil, Australia, New Zealand; and thus, the survey was sent to the respective Toxicology and Pharmacology national associations for these countries. In addition, recipients of the survey were encouraged to forward it to interested parties. The aim was to reach as many scientists within the field as possible, as well as to understand the various geographic locales in which experts reside, so that a network of scientists involved in PBK modelling might eventually be created. However, the authors did not receive feedback on the degree to which the survey was forwarded to other scientists or regulators, and thus, it was not possible to determine the exact number of recipients of this survey.

The answers collected by the EUSurvey tool were exported to a Microsoft Excel file for downstream analysis and interpretation. Data analysis and graphical representation was conducted using Microsoft Excel 2010 (Microsoft Corporation, Redmond, WA, USA). Furthermore, dissemination of the survey replies was elaborated upon by means of an interactive infographic using graphical mapping built by Klimeto (http://klimeto.com/). This visualization approach was chosen to allow for transparency and for sharing of answers in an appealing manner for any parties interested in retrieving such information, while allowing the identities and contact information of the participants to remain anonymous. The interactive infographic was built on Google Chart API as a HTML5 and JavaScript web app, and is currently hosted by the Bolegweb project, (Kliment et al., 2016; http://bolegweb.geof.unizg.hr). The survey results can be accessed at http://bolegweb.geof.unizg.hr/questionaire/pbk/. This paper presents results across the international modelling community, and the web-based results are divided by country. The results are captured in a tabular format and can be visualized by means of pie-charts. For those wishing to access the individual results by country for the PBK model survey using the infographic, use of the Google Chrome browser is advised, and double click on the country of interest, the results will then be displayed. For those using Microsoft Internet Explorer or Mozilla Firefox, if the map does not load, it is recommended to use ctrl+r to refresh. This infographic is also mobile friendly.

## 3 Results

### 3.1 Demographics

**Fig. 2:**
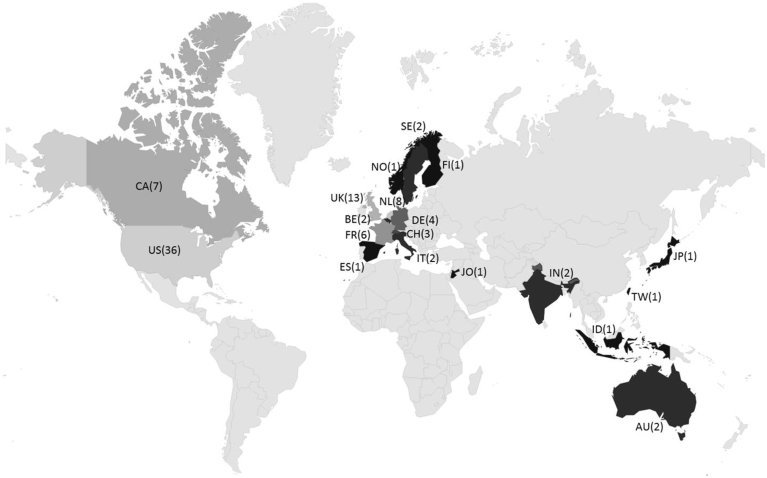
Geographic distribution of the questionnaire results, 93 respondents from 19 countries. Each country is labeled with the ISO 3166 International Standard Country Code, two letters, followed by the number of replies per country in brackets. The results were published and are publicly accessible at the following link http://bolegweb.geof.unizg.hr/questionaire/pbk/, to see individual results double click on a selected country. The color scale represents the lowest number of replies (black) to the highest number (light grey).

A total of 93 survey recipients filled out the online survey from the EUSurvey tool. One recipient preferred not to reply to the survey, due to possible conflicts of interest, but gladly shared experience using PBK models which will be taken into account where appropriate. The 93 replies were received from 19 countries (Fig. 2): Australia (N = 2, 2%), Belgium (N = 2, 2%), Canada (N = 7, 8%), Finland (N = 1, 1%), France (N = 6, 6%), Germany (N = 4, 4%), India (N = 2, 2%), Indonesia (N = 1, 1%), Italy (N = 2, 2%), Japan (N = 1, 1%), Jordan (N = 1, 1%); The Netherlands (N = 8, 9%), Norway (N = 1, 1%), Spain (N = 1, 1%), Sweden (N = 2, 2%), Switzerland (N = 3, 3%), Taiwan (N = 1, 1%), United Kingdom (N = 13, 14%), and the United States of America (N = 35, 38%).

### 3.2 Working sector and professional background

**Fig. 3:**
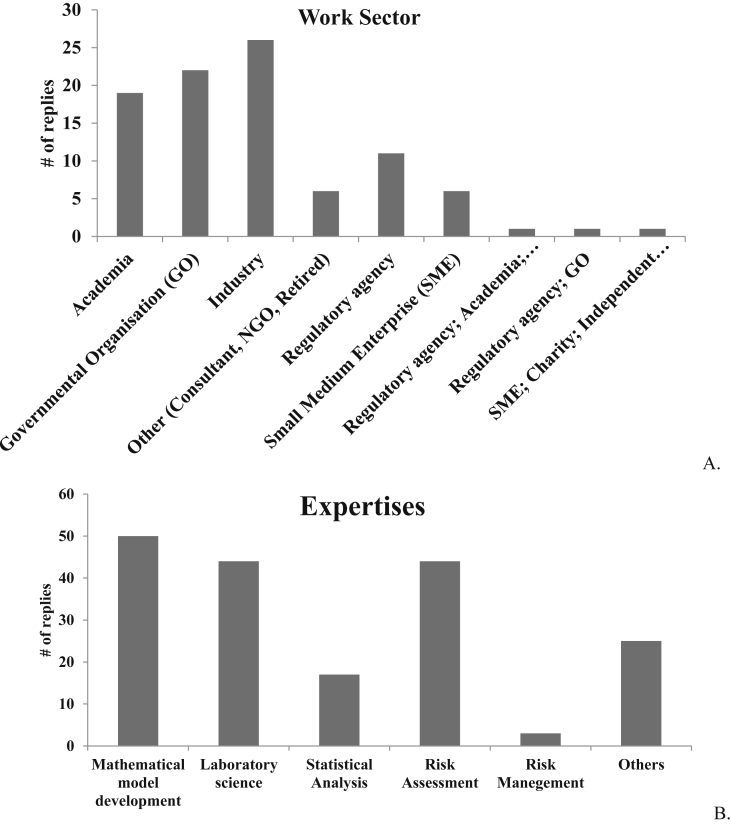
**A.** Woking Sector of the 93 respondents to the survey and **B.** working expertise of the 93 respondents to the survey.

Survey participants represented experts from governmental organizations (N = 22, 24%), industries (N = 26, 28%), academia (N = 19, 20%), regulatory agencies (N = 11, 12%), small medium enterprises (N = 6, 6.5%), and other institutions (N = 6, 6.5%); three participants (3%) declared work in multiple sectors (Fig. 3A). Other sectors identified were: consulting firms, non-government organizations, retired, and a software company. From the industrial sector, nearly half (N = 12, 46%) of participants were in pharmaceutical sector, followed by those in chemical industries (N = 9, 30%), agrochemical (N = 2, 8%), consumer products (N = 2, 8%), cosmetics (N = 1, 4%), and food (N = 1, 4%) sector. From the regulatory agencies, representatives involved in human medicine (N = 7, 44%), chemicals (N = 3,18%), food (N = 3,18%), and environment (N = 3,18%) replied to the survey, in addition to one representative whose work involves regulation of chemicals, food, and human medicine.

The professional background and expertise of the survey participants is summarized in Fig. 3B. Nearly half of the respondents have experience in multiple fields (N = 45), and the most common field of expertise is mathematical model development (N = 50, 27%), followed by risk assessment (N = 44, 24%), laboratory science (N = 44, 24%), statistical analysis (N = 17, 9%), and risk management (N = 3, 2%). Other specific expertise (N = 25, 14%) included molecular modelling, clinical pharmacology, pharmacokinetics, pharmacometrics, physiologically based pharmacokinetic modelling, *in vivo* toxicology, software development, education & mentoring, biopharmaceutics, absorption and formulation effect prediction, epidemiology, regulatory risk assessment, genetic toxicology, food safety, and drug development.

### 3.3 PBK model construction

**Fig. 4:**
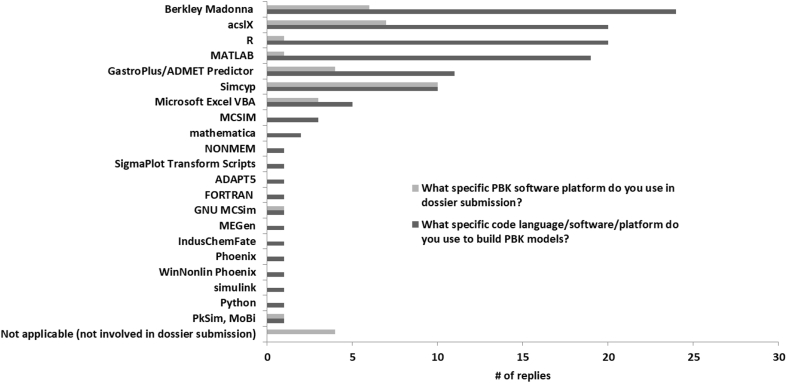
The various language codes, software, or platforms used by survey participants to build PBK models for scientific and regulatory applications.

Of the 93 respondents, 60 (65%) built their own PBK models in-house, and of those, 48 (80%) had experience in coding the models. Of these 48 respondents with coding experience, 21 (44%) followed some guidance documents when building and coding their PBK models. The primary guidance documents followed were the US Environmental Protection Agency's *Approaches for the Application of Physiologically Based Pharmacokinetic (PBPK) Models and Supporting Data in Risk Assessment* (US EPA, 2006) and the World Health Organization International Programme on Chemical Safety's *Characterization and Application of Physiologically Based Pharmacokinetic Models in Risk Assessment* (WHO/IPCS, 2010). Two participants reported that they used the US Food and Drug Administration's (FDA) draft guidance on *Physiologically Based Pharmacokinetic Analyses – Format and Content, Guidance for Industry* (US FDA, 2016) and European Medicines Agency's draft *Guideline on the qualification and reporting of physiologically based pharmacokinetic (PBPK) modelling and simulation* (EMA, 2016). One participant used examples of “best practices” in literature (Loizou et al., 2008, Rietjens et al., 2011), and another used the US FDA's *Guidance for Industry and Other Stakeholders Toxicological Principles for the Safety Assessment of Food Ingredients* (US FDA, 2000). There are 19 programming languages/software platforms reported being used by the respondents (Fig. 4), and the most commonly used programs include Berkley Madonna (Berkeley, CA) (N = 24), acslX (The AEgis Technologies Group, Huntsville, AL) (N = 21), MATLAB (MathWorks, Inc., Natick, MA) (N = 19), and R (N = 18), followed by GastroPlus (Simulations Plus, Lancaster, CA) (N = 11) and Simcyp (Certera, Princeton, NJ) (N = 10). Most participants used multiple platforms.

**Fig. 5:**
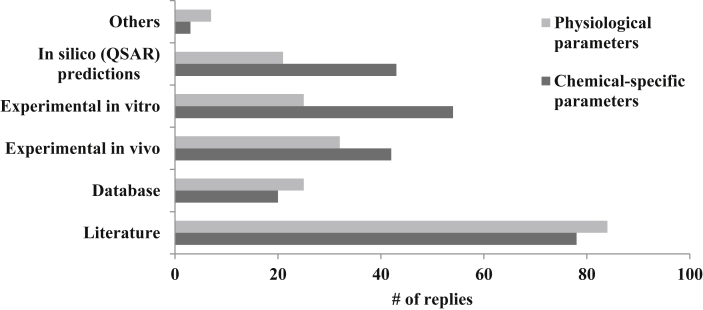
Source of Chemical-Specific and Physiological parameters.

PBK models include both physiological and chemical-specific parameters, and respondents often employ more than one source to obtain model parameter values (Fig. 5). Literature data were found to be the primary sources for obtaining physiological parameters (N = 84, 43%), followed by *in vivo* measurements (N = 32, 16%), databases (N = 25, 13%), *in vitro* measurements (N = 25, 13%), *in silico* predictions (N = 21, 11%), and others (N = 7, 4%). Literature data were also found to be the primary sources for obtaining chemical-specific parameters (N = 78, 33%), followed by *in vitro* measurements (N = 20, 8%), *in silico* predictions (N = 43, 18%), *in vivo* measurements (45%), and databases (22%). Some respondents also provided additional information on databases they used to obtain values for model parameters, and their inputs are summarized in table SM1, in the supplementary material.

**Fig. 6:**
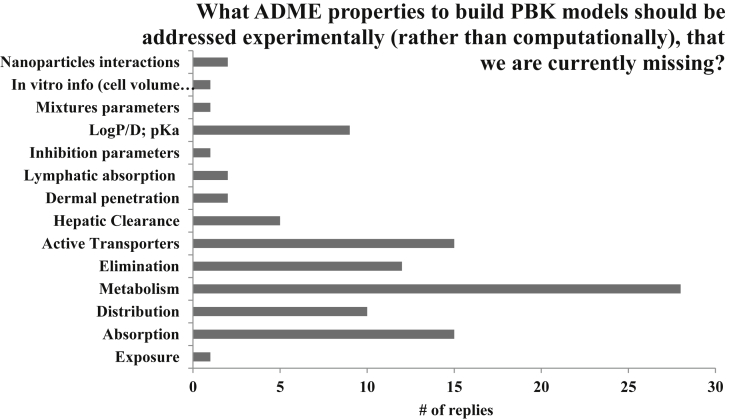
Participants' suggestions of ADME properties that should be derived experimentally when developing PBK models, due to lack of data or information.

Respondents were asked to provide their input as to which ADME properties should be measured using *in vitro* approaches, rather than predicted, when constructing a PBK model. The top responses to this free-text question included metabolism/clearance, active transport, absorption, and chemical properties such as pKa (Fig. 6). In the early stage of model development, *in silico* approaches (e.g., QSAR models) may be used to estimate parameter values that can be replaced later with experimental measurements.

### 3.4 PBK model evaluation

Sensitivity analysis (SA) is an essential step in model validation, as it evaluates the impact of input variations on variability of model outputs. SA can be local or global. A local SA addresses sensitivity related to point estimates of parameter values, while a global SA addresses sensitivity related to distributions of parameter values. For the question of whether SA was conducted as part of the PBK model evaluation routine, 18 (19%) of the respondents answered no. For those who answered yes, most respondents commonly conducted only local SA (N = 32, 34%) or both types of SA (N = 32, 34%), compared to 8 (9%) who conducted only global SA. About 3% of the respondents (N = 3) used other means, such as their own sensitivity metrics. Of interest, one participant noted that most of the models he reviewed did not include SA, even though he believes that SA should be conducted routinely for PBK models that are developed to inform decision-making.

For one of the open questions concerning methods and criteria used to evaluate model performance, 71 (76%) participants responded, and most responses centered around comparing model simulations with observed *in vivo* data such as peak concentration, area under the curve, time to reach peak concentration, and half-life. Various criteria are used to determine goodness of fit. Most participants stated that they visually inspect how well the model fits the data and calculate the fold difference, while others use statistical methods to calculate coefficient of determination, mean absolute percentage error, or root mean-squared error. Respondents also noted that it is important to confirm mass balance and to conduct sensitivity/uncertainty analysis. These individual's answers can be found on the web-based infographic described in methodology section.

For another open question on defining GMP, 66 (71%) respondents answered, and two examples are listed below. The other responses can be found on the web-based infographic.

1. “GMP is less to do with how well a model performs and more to do with how a model is created, and documented and its ability to be reproducible. To me, good modelling practice for PBK modelling means that a model is created in a physiologically meaningful manner using quality and reliable data, with transparency in model structure and parameters and the ability to reproduce simulation results.”
2. “GMP to me includes: transparent declaration of model assumptions; listing available data for model development; choice of appropriate model structure by hypothesis testing; decisions made during model development stated clearly along with their justifications; approaches and results of model performance evaluation reported; both the domain of applicability and inapplicability for the model be identified; model limitations in its application front (for example, data-gap reducing the strength) and the development front (for example, a chance of over parameterization) identified; clear documentation of the code; sensitivity analysis (local and global), uncertainty analysis, and variability analysis conducted; finally, an estimate of overall model confidence metric for its performance.”

### 3.5 PBK model application

**Fig. 7:**
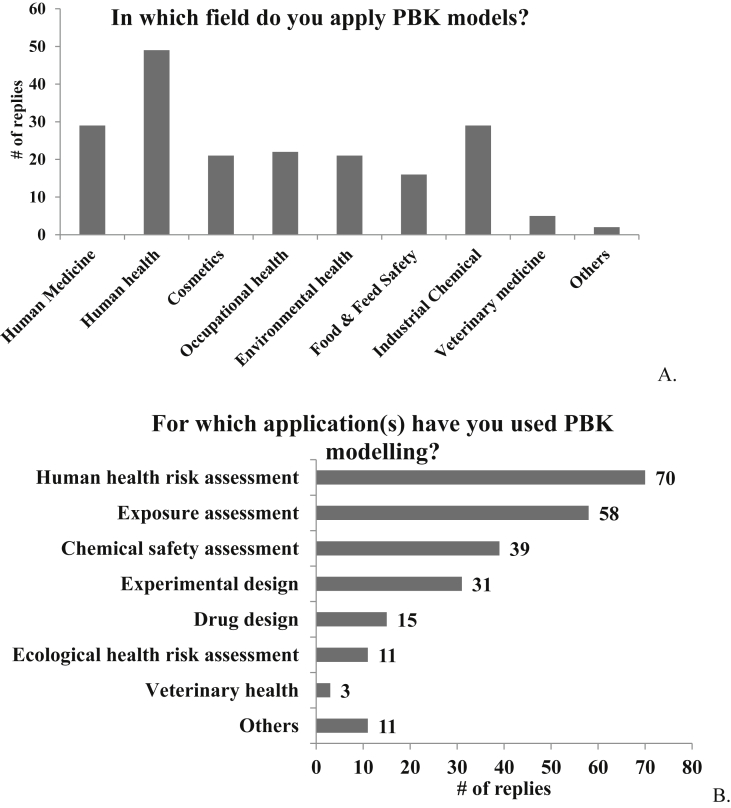
**A.** Selected fields in which participants apply PBK models and **B.** selected applications of PBK models used by participants.

Most participants were found to be involved in PBK modelling from several times a month (N = 28, 30%) to less than once a month but several times a year (N = 32, 34%). About 22 (24%) of the participants incorporated PBK modelling in their daily work, while the remaining participants rarely used PBK modelling all of whom are non-modellers (N = 11, 12%). The primary fields in which the participants apply PBK modelling approaches include human health (N = 49, 25%), human medicine (N = 29, 15%), industrial chemicals (N = 29, 15%), occupational health (N = 22, 11%), environmental health (N = 21, 11%), and cosmetics (N = 21, 11%), (Fig. 7A). Other fields included food & feed safety (N = 16, 8%), veterinary medicine (N = 5, 3%), and others (N = 2, 1%) such as mathematical biology, predictive toxicology, and quantitative systems toxicology (Fig. 7A).

For specific applications, PBK modelling approaches were found mainly to support human health risk assessment (N = 70, 29%), exposure assessment (N = 58, 24%), chemical safety assessment (N = 39, 16%), and experimental design (N = 31, 13%) (Fig. 7B). Applications in drug design, ecological health risk assessment, and veterinary health accounted for 6% (N = 15), 5% (N = 11), and 1% (N = 3), respectively (Fig. 7B). Other (N = 11, 5%) applications of PBK modelling approaches included informing pharmaceutical dose selection, evaluation of pharmaceutical dose adjustment for specific populations, clinical trial design and optimization, drug labelling, and IVIVE to investigate concepts for animal-free risk assessments.

### 3.6 PBK model acceptance by regulatory agencies

Twenty-seven (N = 27, 25%) of the 93 participants declared use of PBK models for submission of dossier/opinions/risk assessment to regulatory agencies, and most of them submitted to agencies in several countries. Submission was done in the following countries: USA (N = 19,40%); Europe (N = 8, 17%); Japan (N = 4, 8%); Australia (N = 3, 6%); Canada (N = 2, 7%); China (N = 2, 7%); The Netherlands, Norway, Spain and UK (N = 1, 2% each); Rest of the World (ROW, N = 1, 2%). Many of those submitting a dossier did so for multiple countries. Nearly half (N = 13, 48%) of the participants submitting a dossier for regulatory purposes using PBK models followed specific guidelines for reporting PBK model development and application. There were two primary sources of guidelines:

1. Report template or internal best practice documents prepared within organizations;
2. Published guidance documents (US EPA, 2006, WHO/IPCS (World Health Organization. International Programme on Chemical Safety), 2010, CEN, 2015);

Of those participants that have experiences submitting PBK models to support regulatory risk assessment, only 39% (N = 36) reported that they have faced challenges in gaining regulatory acceptance of PBK models. Replies can be summarized into the following four main challenges:

i. Lack of expertise in PBK modelling and its applications within the regulatory agencies;
ii. Validation of the model requires testing various scenarios to reassure the agency that due diligence has been undertaken in assessing the model assumptions and predictions, but data for testing are often not available;
iii. Lack of user friendly software for testing the model by risk assessors who are not programmers;
iv. Difference in acceptance criteria between agencies and countries.

Through the International Programme on Chemical Safety (IPCS), the World Health Organization (WHO, http://www.who.int/ipcs/en/) establishes the scientific basis for management of chemicals, and strengthens national capabilities and capacities for chemical safety. A WHO chemical risk assessment network is in place to enhance global efforts to assess the risk to human health from exposure to chemicals by developing tools and guidance documents. A participant reported in our survey that the Chemical-specific Adjustment Factor (CSAF) working group, as part of the WHO Chemical Risk Assessment Network, released a manuscript that discusses recent challenges and experiences in gaining regulatory acceptance of chemical-specific adjustment factors and PBK models (Bhat et al., 2017).

### 3.7 PBK model peer reviewing system

The majority of the participants (N = 83, 89%) agreed that PBK models should be subject to an independent peer review system in order to gain more acceptance in regulatory settings. More than half of the participants (N = 52, 57%) stated that they are willing to act as independent peer reviewers for the regulatory agencies.

## 4 Discussion

The survey concerning applications of PBK models in research and regulatory submission of chemicals was completed by 93 international individuals occupying a wide range of working sectors. The equally high distribution among participants primarily involved human health and exposure assessments using mathematical modelling (N = 50) and risk assessment (N = 42), with nearly half involved in both (N = 22), indicate a balanced set of responses from both model builders and end users. The results provided in this article present a snapshot of the current state of PBK model construction, evaluation, and application.

Although a majority of individuals stated that they use multiple modelling platforms or coding languages, preference of use among sector types was apparent. For example, those involved in academia primarily use Berkeley Madonna and MATLAB, while those associated with government and regulatory agencies primarily use R and acslX, and those in industry primarily use programs such as Simcyp or GastroPlus. Among these listed programs, R is the only open source coding language, and it has the capability to incorporate user-developed packages for a variety of modelling applications. The program acslX provides an intuitive platform that has been used for over 30 years for conducting a wide array of PBK and pharmacodynamic (PD) analyses, but further development and support for this program ceased in November 2016. MATLAB is a computing software that is widely used by scientists and engineers for algorithm development, data analysis, visualization, and numeric computation. In addition to MATLAB, MathWorks Inc. also produces nearly 100 additional products, some of which are ancillary to MATLAB and others that act as stand-alone toolboxes for specialized tasks. Berkley Madonna is known for its ability to rapidly solve differential equations and is widely used amongst the PBK modelling community. These four platforms/languages (R, acslX, MATLAB, and Berkeley Madonna) may be preferred by users in academia or government due to familiarity or costs. In addition, acslX and Berkeley Madonna are used in many PBK modelling training courses, and thus, the comfort level with these four platform types remains high within the PBK modelling community. Familiarity with these platforms also allows peer reviewers, especially those who have attended these training courses but are not programmers, to have the necessary background to review or evaluate models developed using these software programs. On the other hand, models developed using software programs that are unfamiliar to reviewers may not be properly vetted.

Both Simcyp and GastroPlus are proprietary programs that offer numerous options for model parameterization (e.g., built-in *in silico* models or user-specified input) in a user-friendly graphical interface. These programs are primarily used within the pharmaceutical industry because screening-level models can be developed much more quickly through the menu choices, default settings, and internal algorithms for model parameterization. Interestingly, Simcyp was the top platform used for dossier submission to regulatory agencies as reported by the participants. This result may be due to several regulatory agencies, including the US FDA, the European Medicines Agency (EMA), and Japanese Pharmaceuticals and Medical Devices Agency, being affiliates of the Simcyp Consortium, which is a collaborative research centre for PBK and mechanistic modelling. Another unique aspect of these two generic PBK modelling platforms is that the proprietary nature of drug development processes necessitates a formal agreement between the pharmaceutical companies and software developers to allow those software developers to use confidential preclinical and clinical data provided by the companies for supporting software development. At the same time, the software developers ensure the software products are relevant to the needs of companies, such as providing recommendations on dosing regimens or formulations, examining drug-drug interactions, or designing clinical trials.

In addition to modelling platform uses, the survey also gathered information regarding the sources for obtaining values of model parameters. Physiological parameters such as body weight, organ weight, cardiac output, respiratory parameters, and milk secretion were found to be primarily retrieved from the literature and from *in vivo* studies. Chemical-specific parameters (e.g., metabolism rate, absorption rate, partition coefficient, fraction unbound in plasma) or properties that are used to estimate these parameters (e.g., polarity, pH, log K_ow_) were also found mostly to be taken from the literature. Secondary sources of chemical-specific data included *in silico* predictions, which were primarily used by those in academic and governmental sectors; or *in vitro* measurements, which were primarily used by those in the industry sector. Such differences may be due to industries having more resources to conduct *in vitro* experiments, compared with academia or government agencies that have limited resources. An excellent summary of these various approaches for model parametrization can be found in Bessems et al. (2014). A list of available databases reporting parameters for building PBK models provided by the respondents of the survey is available in the supplementary material. Although several of these databases are open source, most require a license for access. Some participants indicated that data required to fully understand ADME properties is sorely lacking, as is data required to understand the physiology of cells (e.g., volumes, diameters, etc.). Other participants suggested that clinical data may act as appropriate sources for examining the limitations of IVIVE.

Often, it is the modeller's purpose to build the most parsimonious or simplest model for the data at hand. Traditionally, this approach required optimizing certain parameters so that model outputs agree with available *in vivo* data. Usually, these parameters are related to characteristics that impact entry or disappearance of the chemical in plasma (i.e., absorption or metabolism/excretion), and simply optimizing these parameters in accordance with allometric scaling would often be sufficient for model evaluation. However, as the number of chemicals in commerce has continued its precipitous rise (Binetti et al., 2008, Egeghy et al., 2012), the limitations of this approach have become obvious. Specifically, it is often the case that most chemicals do not have *in vivo* PK data available for comparing against model predictions. Moreover, policies in the US and EU are shifting towards reductions in animal studies; a good example is the full ban of testing cosmetic ingredients and final cosmetic products in Europe, in force since 2013. In these cases, model development and evaluation must rely on better mechanistic understanding of ADME properties, as well as NAMs to derive values for model parameters required to describe these ADME properties. It was found from the survey that, among different NAMs, *in vitro* methods are often preferred over *in silico* methods. This result could be explained by a higher degree of confidence that *in vitro* measurements may confer over *in silico* predictions, in addition to the need of having measurement data for training *in silico* models.

There is universal consensus among individuals and working sectors that metabolism and clearance properties should be derived experimentally. Those participants in both government agencies and academia expressed a second priority to experimentally measure partition coefficients, followed by transporter processes. Alternatively, those in industry consider transporter processes to be a secondary focus, followed by partition coefficients. Regardless, it is apparent that for greater acceptance of models by regulatory agencies, the properties of metabolism, partitioning, and transport are of high priority.

PBK models developed for pharmaceutical products focus primarily on the fate of the parent compound, along with its distribution in the body and arrival at the site of action; thus determining an appropriate clearance value is essential to achieve these goals. On the other hand, metabolism is key in understanding mode of action for environmental chemicals, and availability of data on kinetic constants (*V*_max_ and *K*_*m*_) is essential. Several efforts are underway to map exposure knowledge (Teeguarden et al., 2016) and adverse outcomes (Ankley et al., 2010; https://aopwiki.org/wiki/) with high throughput *in vitro* dose-response data to significantly expand our understanding of chemical fate and effects (Wittwehr et al., 2017). However, little attention has been placed on experimental identification of metabolites to enable testing of their ability to trigger adverse outcome pathways. Quantification of the generation and clearance rates of these metabolites can be accomplished *in vitro* through whole-cell experiments, or through studies on individual phase I and phase II microsomal or cytosolic enzymes. Measuring chemical responses upon interaction with the individual enzymes can allow for determining kinetic constants like *V*_max_ and *K*_*m*_ for different transformation pathways (e.g., oxidation, hydroxylation, epoxidation). Enzyme fractions can be purchased for different organs (e.g., liver, lungs, kidney, skin), and the complexity of PBK models will vary as a function of the number of metabolites accounted for, the number of organs compartmentalized, and differences between and within species (Punt et al., 2008, Punt et al., 2009, Punt et al., 2016). Overall, the advancement in these *in vitro* technologies are contributing to generation of biotransformation data (e.g., *V*_max_ and *K*_*m*_) necessary for the proper function of PBK models.

Most respondents agreed that GMP should be followed during model evaluation (EFSA, 2014, Loizou et al., 2008). GMP involves a combination of several considerations: (1) a transparent description of how the model is created; (2) appropriate documentation, including model assumptions, sensitivity analysis (local and global), uncertainty analysis, and variability analysis; and (3) an estimate of overall model performance and reproducibility. These considerations were reiterated at an EMA workshop (London, UK, November 2016) in which participants discussed the key elements that should be included in a draft document on qualification and reporting of PBK analyses. During the EMA workshop, the need for SA was central to the discussion, as some attendees debated the need for a global SA when using a commercial PBK platform to develop a PBK model, and that focus should be placed on parameters with less uncertainty (Zhao, 2017).

Currently, most evaluation of PBK models is conducted by visually comparing model predictions with experimental data, such as area under the curve (AUC), peak concentration (*C*_max_), time to peak concentration (*t*_max_), and plasma half-life. Besides visual examination, other criteria are used to examine goodness of fit, such as setting error thresholds of 0.5–2-fold, or even 0.8–1.25-fold. Pharmaceutical companies often use this latter threshold in their determination of the bioequivalence between two drugs. In other words, bioequivalence in this case is defined to be no more than a 20% difference between the AUC and *C*_max_ of two medicines, usually a generic medicine compared to the innovator medicine (MedSafe, 2015, Bpac, 2009).

Only half of the participants who have experience in submitting PBK models for regulatory agencies review stated that they used any sort of guidelines when developing or reporting their models. Since most guidelines generally contain common modelling practices, even though survey participants and many modellers may not explicitly follow published guidelines, it is likely the case that their familiarity with modelling etiquette has resulted in their adhering to a majority of the guidelines. Despite a lack of clearly established GMP guidelines, certain pharmaceutical and industrial sectors are collaborating with regulatory agencies on a more frequent basis in an effort to apply NAMs and PBPK modelling approaches in evaluation of their products’ safety and to receive permission to be released onto the market. For example, in 2015, the results of a survey focusing on preclinical pharmacokinetic/pharmacodynamics (PK/PD) revealed that pharmaceutical companies have increased the use of preclinical PK/PD analysis in all therapeutic areas (Schuck et al., 2015). This trend is reflected through an increasing number of submissions containing PBK model analysis to regulatory agencies, in the US to FDA and in Europe to EMA (Rowland et al., 2015). More specifically, PBK models are applied to support benefit–risk evaluations by providing a mechanistic basis for extrapolation beyond the clinical trial population, by reducing uncertainty, and by predicting drug–drug interactions (DDIs) in specific groups of populations (e.g., elderly, pediatric) (Shepard et al., 2015). Furthermore, PBK models are developed within a systems pharmacology context to efficiently extrapolate PK/PD properties in various healthy and patient populations (Jamei, 2016), for both adults and children, for better precision medicine (Wagner et al., 2015). Studies have shown that application of PBK models has the potential for improving pediatric drug trials (Leong et al., 2012) and oncology treatments (Yoshida et al., 2017). EMA and the US FDA both released draft guidance intended to help pharmaceutical companies to report PBK model analyses to the respective regulatory agency in a harmonized format (EMA European Medicine Agency, 2016, US FDA, 2016). These more recent movements show that there is an increasing interest in applications of PBK models in several branches of science (medicine, pharma, environmental, exposure) and that these models may gain more regulatory acceptance.

Perhaps it is the combination of a lack of established standardized GMP guidelines and the wide range of computing platforms used for model development that serve as the two greatest obstacles for PBK model use in regulatory risk assessment. The unique needs of each regulatory agency add further difficulty in establishing GMP guidelines that can be used universally in risk assessment. In most cases, a standard checklist to evaluate the quality of a PBK model and the appropriateness of its applications is unavailable for decision makers who possess little knowledge of the PBK modelling process. Establishment of GMP across the modelling community can act as an excellent start for developing such a standardized checklist among scientific peers, which can then be communicated to and adapted by regulatory agencies. Over time, these agencies can revise the checklist to meet their specific needs, while still maintaining the standardized approach for reviewing model development and evaluation that are generalizable among different regulatory agencies. The lack of a common set of computing platforms for PBK modelling, especially in the field of environmental health, has been a challenge for many years. Harmonizing the general structure of model code among different platforms can serve as an initial step to address this issue. The programming command and code sorting will certainly remain unique within each platform, but the general structure for coding differential equations and organizing various sections of the model (e.g., defining physiological and chemical-specific parameters separately) tend to be conducive to forming a template that can be easier to understand by reviewers who are not familiar with a specific software.

The PBK models published by academia and industry often are developed in an attempt to seek an answer to a specific scientific question related to a certain chemical or chemicals (e.g., interpreting biomarker data, organizing animal time-concentration data), and the purpose of such a study can differ significantly from that of a regulatory agency drafting safety guidelines for those chemicals. In many cases, it may be perfectly suitable to use a PBK model to help answer these scientific questions through various academic exercises, with some supporting *in vivo* data to verify such predictions. However, when the purpose is to use a PBK model to support regulatory decision-making, the model requires careful vetting of selected parameter values, model code and structure, and methods of model evaluation, before confidence can be gained in applying that model for regulatory risk assessment purposes.

In addition to proper documentation and quality control, the validity of a model and its application heavily relies on attaining the proper data to derive the most appropriate values for model parameters. Over time, the advancements in molecular biology, *in vitro* testing systems, and *in silico* techniques have led to generation of new knowledge regarding biological systems, in addition to data the necessary for facilitating more mechanistic-based model development and improving a model's capability to predict and extrapolate. Inclusion of transporter processes and -omics data will have the potential to further improve confidence in model parameters (Poulin et al., 2016). The modelling community should be aware that although these newer generation PBK models may possess increased predictive capability due to inclusion of mechanistic processes and emerging data, additional challenges are introduced to risk assessors attempting to review these more detailed and complex models in support of regulatory decision making. Especially in the case which experimental *in vivo* data are not available for comparison to model predictions, regulatory agencies are likely to remain reluctant to involve such models in their chemical safety assessments, highlighting a need to develop new guidance documents to help regulatory agencies in reviewing and accepting these type of models.

## 5 Conclusions

This paper describes how PBK models are currently constructed, evaluated, and applied in various working sectors and countries. This analysis provides insight into the current state of the PBK modelling and user community, in addition to a cursory contact list of modellers available for peer review. Several limitations of acceptance of PBK models into the regulatory risk assessment process still remain due to lack of guidance, lack of data, or lack of expertise. However, the modelling community is continuously expanding, and with it, PBK models are gaining ground for use in various scientific and regulatory risk assessment applications. A similar PBK modelling survey should be updated periodically to keep pace with evolving databases, available software, and new challenges.

## Disclaimer

The U.S. Environmental Protection Agency has provided administrative review and has approved this paper for publication. The views expressed in this paper are those of the authors and do not necessarily reflect the views of the U.S. Environmental Protection Agency or the European Commission.

## Appendix A Supplementary data

Supplementary material_V2 Supplementary material_V2

## Footnotes

1 The term PBK model is synonymous with physiologically based pharmacokinetic (PBPK), physiologically based biokinetic (PBBK), and physiologically based toxicokinetic (PBTK) models.

2 http://publications.jrc.ec.europa.eu/repository/bitstream/JRC96418/eurl%20ecvam%20toxicokinetics%20strategy.pdf.
