# Supplementary material for: Investigating the state of physiologically based kinetic modelling practices and challenges associated with gaining regulatory acceptance of model applications
Source: Regul Toxicol Pharmacol. 2017 Nov;90:104–15. doi: 10.1016/j.yrtph.2017.08.019 (PMC5656087; doi:10.1016/j.yrtph.2017.08.019)
Supplement: Supplementary material_V2 [file mmc1.docx]

**Supplementary material**

**Investigating the state of the physiologically based kinetic modeling practices and challenges associated with gaining regulatory acceptance of model applications**

Alicia Paini^1^, Jeremy A. Leonard^2^, Tomas Kliment^3^, Yu-Mei Tan^4^, Andrew Worth^1^

^1^Directorate Health, Consumers and Reference Materials, European Commission, Joint Research Centre, Via E Fermi 2749, 21027, Ispra, Italy.

^2^Oak Ridge Institute for Science and Education, Oak Ridge, TN 37831, USA

^3^Klimeto, Juzna 4, Roznava, Slovakia.

^4^U.S. Environmental Protection Agency, National Research Laboratory, Research Triangle Park, NC 27709, USA.

* Corresponding author at: [alicia.paini@ec.europa.eu](mailto:Alicia.paini@ec.europa.eu)

European Commission, Joint Research Centre, Directorate F – Health, Consumers and Reference Materials, Chemical Safety and Alternative Methods Unit, Via E. Fermi 2749, TP 126, I-21027 Ispra (VA), Italy.

**1. EUSurvey full questionnaire:**

Applications of Physiologically Based Kinetic (PBK) models in science and regulatory submission.

Fields marked with * are mandatory.

Scope of the Survey

Welcome to the EURL ECVAM PBK model survey!

Thank you for taking time to take part to this survey!

Today we will ask you 18 questions, 14 with YES/NO or multiple choice answers and 4 in free text format. The YES/NO questions might expand to break-up questions, depending on your reply, and in certain cases also allow you to add comments in free text. In total the survey should take 5 - 10 min.

The aim of this survey is to understand frequency of use and applications of Physiologically Based Kinetic (PBK) models (PBK is a terminology synonymous of PBPK/PBTK/PBBK, so PBK = PBPK/PBTK/PBBK) in science and regulatory submission.

Download privacy statement

[Specific_Privacy_Statement_PBK.pdf](https://ec.europa.eu/eusurvey/files/57529ca8-e8f3-47d8-b3b7-b86bbf105620)

*****I have read and accept the terms of the privacy statement

|  | YES |
| --- | --- |

Your Details

Please provide your name and surname

Questions

*****1. In which sector do you work?

|  | Industry |
| --- | --- |
|  | Regulatory agency |
|  | Academia |
|  | Small Medium Enterprise |
|  | Governmental Organisation |
|  | Other |

***** 2. In which country do you currently work?

3. What kind of work do you do?

|  | Mathematical model development |
| --- | --- |
|  | Laboratory science |
|  | Risk assessment |
|  | Risk management |
|  | Statistical analysis |
|  | Others |

*****4. How often do you use PBK model in your work?

|  | Daily |
| --- | --- |
|  | Several times a month |
|  | Less than once a month but several times a year |
|  | Once every few years |
|  | Used in the past/I do not use them |

5. For which application(s) have you used PBK modelling?

|  | Human health risk assessment |
| --- | --- |
|  | Ecological health risk assessment |
|  | Chemical safety assessment |
|  | Veterinary health |
|  | Experimental design |
|  | Drug design |
|  | Exposure assessment |
|  | Others |

6. In which field do you apply PBK models?

|  | Human medicine |
| --- | --- |
|  | Veterinary medicine |
|  | Food & Feed Safety |
|  | Industrial Chemical |
|  | Cosmetics |
|  | Human health |
|  | Occupational health |
|  | Environmental assessment |
|  | Others |

***** 7. Do you build your own PBK models?

|  | YES |
| --- | --- |
|  | NO |

8. Which sources do you mainly use to obtain chemical-specific parameters (e.g., partition coefficients, metabolism, skin/oral absorption, protein binding) for the model (e.g., in vitro system, in silico predictions, database)?

|  | Literature |
| --- | --- |
|  | Database |
|  | Experimental in vivo |
|  | Experimental in vitro |
|  | In silico (QSAR) predictions |
|  | Others |

9. What ADME properties to build PBK models should be addressed experimentally (rather than computationally), that we are currently missing?

10. Do you know or use any database providing ADME properties?

11. Source of the physiological parameters

|  | Literature |
| --- | --- |
|  | Database |
|  | Experimental in vivo |
|  | Experimental in vitro |
|  | In silico (QSAR) predictions |
|  | Others |

***** 12. Do you perform uncertainty analysis/sensitivity analysis?

|  | YES |
| --- | --- |
|  | NO |

13. How do you evaluate model performance? What methods and criteria do you use?

14. What does "GOOD MODELLING PRACTICE" means to you

*****15. Have you used PBK models for submission of dossiers/opinions/risk assessment to regulatory agencies?

|  | YES |
| --- | --- |
|  | NO |

***** 16. Do you have any challenges and experiences to share in gaining regulatory acceptance of PBK models?

|  | YES |
| --- | --- |
|  | NO |

*****17. Should PBK models be subject to independent peer review in order to be used in Risk Assessment?

|  | YES |
| --- | --- |
|  | NO |

*****18. Would you like to participate as an independent peer reviewer of PBK models?

|  | YES |
| --- | --- |
|  | NO |

2. Table SM 1. List of available databases reported in the EUsurvey for building PBK models.

| **Chemical Specific Parameters** | | |
| --- | --- | --- |
| **Name** | **Link** | **Availability** |
| **For marketed drugs:** | | |
| US FDA drug database - drugs@fda | <https://www.fda.gov/drugs/informationondrugs/ucm135821.htm> | **Open source** |
| DIDB from U-Washington (Seattle, WA, USA) | <https://www.druginteractioninfo.org/> | **Licence** |
| Drugbank | <https://www.drugbank.ca/> | **Open source** |
| Pharmapendium | <https://www.pharmapendium.com/#/login> | **Licence** |
| Merck Index | <https://www.rsc.org/merck-index> | **Licence** |
| GastroPlus  ADMET predictor | <http://www.simulations-plus.com/software/gastroplus/>  <http://www.simulations-plus.com/software/admet-property-prediction-qsar/> | **Licence** |
| Simcyp | <https://www.certara.com/software/pkpd-modeling-and-simulation/physiologically-based-pharmacokinetic-modeling-and-simulation/> | **Licence** |
| Simcyp free ADME calculator app | <https://play.google.com/store/apps/details?id=air.android.com.simcyp.calculators&hl=it> | **Open source** |
| PopGen | <http://xnet.hsl.gov.uk/popgen/> | **Open source** |
| **For more general environmental chemicals:** | | |
| US EPA iCSS dashboard | <https://actor.epa.gov/dashboard/> | **Open source** |
| PubChem Compound | <https://pubchem.ncbi.nlm.nih.gov/search/> | **Open source** |
| The  Interspecies database – | <https://www.interspeciesinfo.com/> | **Open source** |
| ChemSpider | <http://www.chemspider.com/> | **Open source** |
| EDETOX database for dermal penetration data | <https://apps.ncl.ac.uk/edetox/> | **Open source** |
| US EPAs ECOTOX database | <https://cfpub.epa.gov/ecotox/> | **Open source** |
| ToxCast and Tox21 datasets | <https://www.epa.gov/chemical-research/toxicity-forecaster-toxcasttm-data> | **Open source** |
| Httk | <https://cran.r-project.org/web/packages/httk/index.html> | **Open source** |
| on-line chemical modelling environment -oCHEM | <https://ochem.eu/home/show.do> | **Open source** |
| OECD toolbox | <http://www.oecd.org/chemicalsafety/risk-assessment/oecd-qsar-toolbox.htm> | **Open source** |
| Episuite | <https://www.epa.gov/tsca-screening-tools/epi-suitetm-estimation-program-interface> | **Open source** |
| **Physiological Parameters** | | |
| **Name** | **Link** |  |
| Embedded in Simcyp | <https://www.certara.com/software/pkpd-modeling-and-simulation/physiologically-based-pharmacokinetic-modeling-and-simulation/> | **Licence** |
| Implemented in PkSim | <http://www.systems-biology.com/products/PK-Sim.html> | **Licence** |
| Built in Gastroplus ADMET predictor | <http://www.simulations-plus.com/software/gastroplus/>  <http://www.simulations-plus.com/software/admet-property-prediction-qsar/> | **Licence** |
| UK Census | <https://www.ukcensusonline.com/> | **Open source** |
| Child growth graphs | <https://www.cdc.gov/growthcharts/cdc_charts.htm> | **Open source** |
| ICRP | <http://www.icrp.org/page.asp?id=145> | **Open source** |
| MEGen | <http://megen.useconnect.co.uk/> | **Open source** |
| US EPA Physiological Information Database  PID database  HERO Database | <https://cfpub.epa.gov/ncea/risk/recordisplay.cfm?deid=202847&CFID=90333472&CFTOKEN=83385957>  <https://hero.epa.gov/hero/index.cfm> | **Open source** |
| RIVM Interspecies database | <https://www.interspeciesinfo.com/> | **Open source** |
| P3M | Price et al., (2003) Modeling interindividual variation in physiological factors used in PBPK models of humans. Crit Rev Toxicol 33(5):469-503. | **Literature** |
| NHANES | <https://www.cdc.gov/nchs/nhanes/> | **Open source** |
| Brown et al, 1997 | Brown et al., 1997 (Toxicol. Indust. Health 13:407-484) | **Literature** |
| PhysioBank | <https://www.physionet.org/physiobank/database/> | **Open source** |
| HESS | <http://www.nite.go.jp/en/chem/qsar/hess-e.html> | **Upon registration** |
